# Triazophos (TAP) removal in horizontal subsurface flow constructed wetlands (HSCWs) and its accumulation in plants and substrates

**DOI:** 10.1038/s41598-017-05874-0

**Published:** 2017-07-14

**Authors:** Juan Wu, Zhu Li, Liang Wu, Fei Zhong, Naxin Cui, Yanran Dai, Shuiping Cheng

**Affiliations:** 1 Tongji University, Laboratory of Yangtze River Water Environment, Ministry of Education, Shanghai, 200092 PR China; 2 Hubei University of Arts and Science, Hanjiang Institute, Xiangyang, 441053 PR China; 3 Nantong University, Nantong, Jiangshu 226007 PR China; 4 Institute of Hydrobiology, Chinese Academy of Sciences, Wuhan, 430072 PR China

## Abstract

Triazophos (TAP) is a widely used phosphorus pesticide in China that possesses a potential risk for water pollution. We have studied the removal efficiency of TAP using pilot-scale horizontal subsurface flow constructed wetlands (HSCWs) as well as the contribution of plants, substrates and other pathways to its removal. For TAP concentrations of 0.12 ± 0.04 mg L^−1^, 0.79 ± 0.29 mg L^−1^ and 3.96 ± 1.17 mg L^−1^, the removal efficiencies were 94.2 ± 3.7%, 97.8 ± 2.9% and 84.0 ± 13.5%, respectively, at a hydraulic loading rate (HLR) of 100 mm d^−1^; at an HLR of 200 mm d^−1^, the removal efficiencies were 96.7 ± 1.3%, 96.2 ± 1.7% and 61.7 ± 11.1%, respectively. The isopleth maps of TAP along the direction of flow indicate that most of the TAP removal occurred in the front and middle regions, while the major removal region would move forward with increasing influent TAP. Plant and substrate accumulation accounted for 0.035 ± 0.034% and 4.33 ± 0.43% of the total removal, respectively, indicating that over 95% of the TAP removal was achieved through other mechanisms. Thus, these results suggest HSCWs can be an effective approach with which to treat TAP contaminated water. Furthermore, the longitudinal scale and hydraulic conditions, as well as the roles of plants, substrates and microbes and their interactions, should be further considered in the design and application of CWs for pesticide pollution control.

## Introduction

Triazophos (O, O-diethyl-O-(1-phenyl-1,2,4-triazole-3-base) sulfur phosphate, C_12_H_16_N_3_O_3_PS, TAP), a moderately toxic organophosphorus pesticide (OPP)^1^, is widely used in agricultural and aquaculture activities in China^2^. However, the widespread application of TAP represents a risk to human health as well as the ecological system due to its high chemical and photochemical stability. In its applications to agriculture and aquaculture, TAP will diffuse throughout surface and ground water with a half-life of 25.4 days in canal water under Indian climatic conditions^3^. According to the US EPA ECOTOX databases, TAP may harm non-target aquatic and terrestrial organisms, and it may threaten human health through the food chain (https://cfpub.epa.gov/ecotox/). TAP can cause acute and chronic problems i aquatic organisms^2, 4, 5^, and its residue in fruits and vegetables poses a dietary risk to human life^6^.

Constructed wetland (CW), which is an eco-friendly and low-cost water treatment technology, has been successfully applied for the treatment of surface and ground water that has been contaminated by OPPs^7, 8^. TAP is removed through several pathways in the CWs, including through uptake and degradation by plants, adsorption by substrates, degradation by microbes, and other degradation mechanisms such as volatilization, hydrolysis, photolysis, and so on ref. 9. The complex interactions between plants, substrates and microbes in the CWs makes it challenging to understand and predict the efficiency and mechanism of pesticide removal in CW applications.

Our previous studies, which were conducted using different plant species to investigate the effects of phytoremediation on TAP^10–12^, collectively evaluated the removal efficiencies of plants and microbes in hydroponic systems. However, the performance and contributions of the components of CWs (plants, substrates, microbes, etc.) to the removal of TAP are still inadequately understood. In the present paper, three sets of pilot-scale horizontal subsurface flow constructed wetlands (HSCWs) were employed to monitor the corresponding removal efficiency and to investigate the fate of TAP in the HSCWs.

## Results

### The removal efficiency of TAP

**Figure 1:**
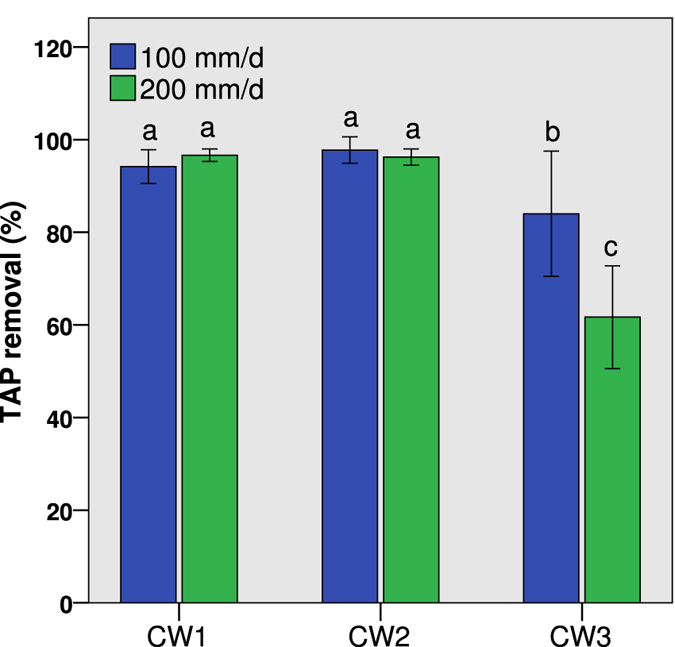
TAP removal efficiencies in the horizontal flow constructed wetlands (HSCWs) fed with mean influent TAP concentrations of 0.12 ± 0.04 mg L^−1^ (CW1), 0.79 ± 0.29 mg L^−1^ (CW2) and 3.96 ± 1.17 mg L^−1^ (CW3) at HLRs of 100 mm d^−1^ and 200 mm d^−1^. Different letters indicate significant differences among the columns (P < 0.05).

At an HLR of 100 mm d^−1^, the mean removal efficiencies of TAP were 94.2 ± 3.6%, 97.8 ± 2.9% and 84.0 ± 13.5% in CW1, CW2 and CW3 (n = 6), respectively; at an HLR of 200 mm d^−1^, mean removal efficiencies of TAP were 96.7 ± 1.3%, 96.2 ± 1.7% and 61.7 ± 11.1% in CW1, CW2, and CW3 (n = 5), respectively (Fig. 1). A two-way ANOVA indicated that both the HLR and the influent TAP concentration had significant effects on TAP removal (P < 0.01), whereas the difference in the removal between CW1 and CW2 is not notable (P = 0.59). The TAP removal efficiency differed markedly between the two HLRs; there was a higher mean efficiency of 92.0 ± 9.8% at and HLR of 100 mm d^−1^ relative to 84.9 ± 18.0% at an HLR of 200 mm d^−1^. Compared to the stable performances of TAP removal in CW1 and CW2 at 100 mm d^−1^, the TAP removal percentages in CW3 displayed a downward trend from 98.0% to 72.4% when the influent TAP concentration increased from 2.68 mg L^−1^ in September to 5.25 mg L^−1^ in March.

### The longitudinal distribution of TAP in the substrates

**Figure 2:**
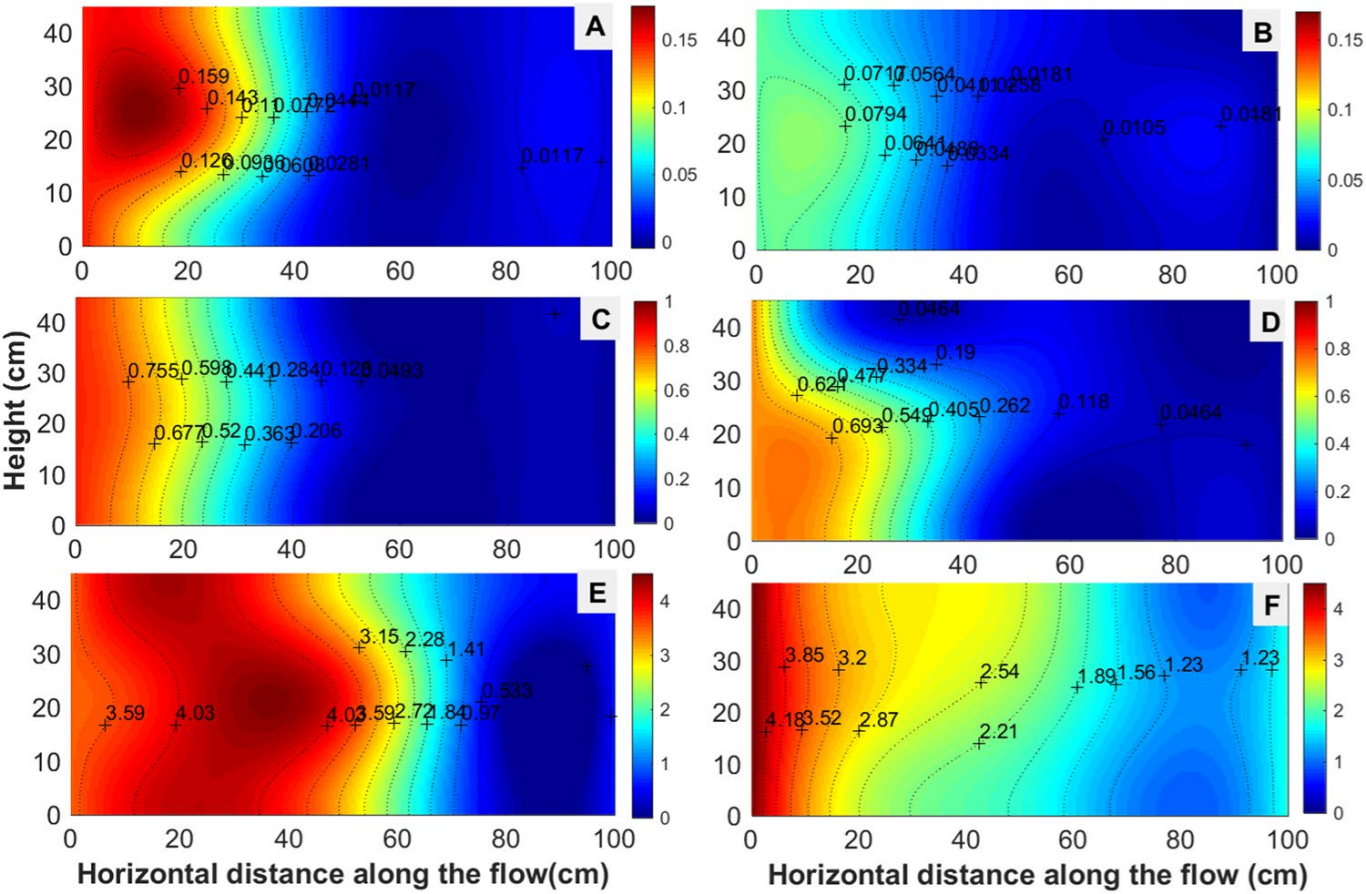
Longitudinal isopleth maps of the TAP concentrations in the horizontal flow constructed wetland (HSCW) loaded with influent TAP concentrations of 0.12 ± 0.04 mg L^−1^ (**A** and **B**), 0.79 ± 0.29 mg L^−1^ (**C** and **D**) and 3.96 ± 1.17 mg L^−1^ (**E** and **F**) at the hydrological loading (HLR) of 100 mm d^−1^ (**A**, **C** and **E**) and 200 mm d^−1^ (**B**,**D** and **F**). The legend bar on the right side of each plot was scaled with the range of the TAP concentration.

Lengthwise isopleth maps of the TAP concentrations in the HSCWs visually illustrate clear longitudinal dispersion trends of TAP corresponding to different influent concentrations at the two HLRs (Fig. 2). Generally, the isolines are roughly vertical and parallel to one another, indicating that the TAP was gradually removed along the flow direction. In addition, the denser isolines in the left half part of the CWs indicate that most of the reduction in the TAP concentration occurred in the front region, while the removal of TAP in the second half of the region was notably mild. At an HLR of 100 mm d^−1^, 70.24% of TAP removal was accomplished in Zones I and II, wherein the removal was approximately 87% at 200 mm d^−1^. Compared to CW1 and CW2, the major purification zone extended to Zone III in CW3, especially at 100 mm d^−1^, implying that the primary TAP purification area will migrate forward along the flow with increasing influent TAP concentrations. The TAP removal patterns significantly differed between the two HLRs, with a faster reduction at 200 mm d^−1^ relative to that at 100 mm d^−1^.

### TAP accumulation in plants and substrates

**Figure 3:**
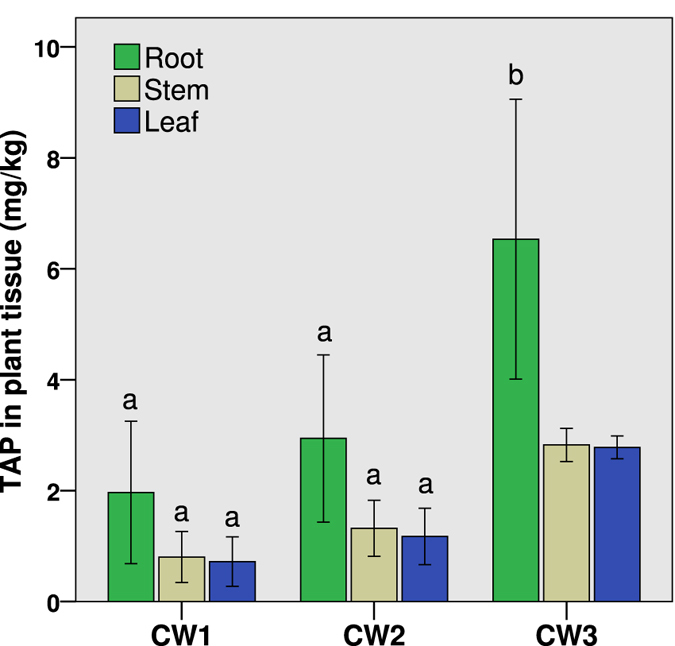
TAP content in tissues of *Canna indica* in the horizontal flow constructed wetlands (HSCWs) fed with mean influent TAP concentrations of 0.12 ± 0.04 mg L^−1^ (CW1), 0.79 ± 0.29 mg L^−1^ (CW2) and 3.96 ± 1.17 mg L^−1^ (CW3) at the hydraulic loading of 100 mm d^−1^. Different letters indicate significant differences among the columns (P < 0.05).

The TAP content in tissues of *C. indica* from the front, middle and rear regions of the CWs were considered in triplicate, and then mean values were calculated (n = 3, Fig. 3). The TAP accumulated in the roots was considerably higher than in the stems and leaves (P < 0.01), wherein the mean TAP concentration in the roots was 3.81 ± 2.63 mg kg^−1^, nearly 2.5 times that in the other tissues. However, the differences between the TAP concentrations in the stems and leaves were not significant (P > 0.05). The influence of the influent TAP concentration on its content in tissues was significant (P < 0.01), with the highest mean content of 4.05 ± 2.26 mg kg^−1^ observed in CW3. Meanwhile, the mean content in CW1 (1.16 ± 0.94 mg kg^−1^) was similar to that in CW2 (1.81 ± 1.19 mg kg^−1^). The correlation between the TAP content in the tissues and the TAP concentration in water from the sampling ports indicated that the accumulation of TAP in the plant tissues was closely correlated with the TAP in the regions from which the plants were collected.

**Figure 4:**
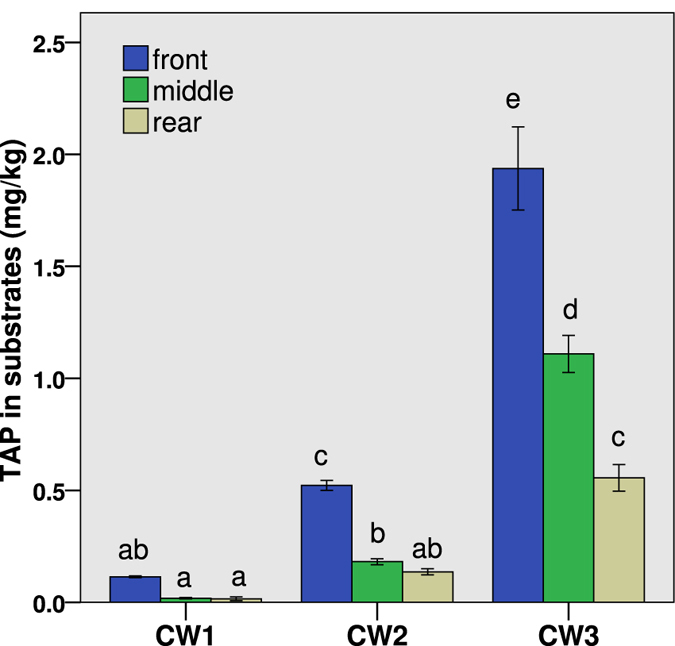
TAP content in the substrates from different regions (the front, middle and rear regions along the direction of flow) of the horizontal flow constructed wetlands (HSCWs) fed with mean influent TAP concentrations of 0.12 ± 0.04 mg L^−1^ (CW1), 0.79 ± 0.29 mg L^−1^ (CW2) and 3.96 ± 1.17 mg L^−1^ (CW3) at the hydraulic loading of 100 mm d^−1^. Different letters indicate significant differences among the columns (P < 0.05).

**Figure 5:**
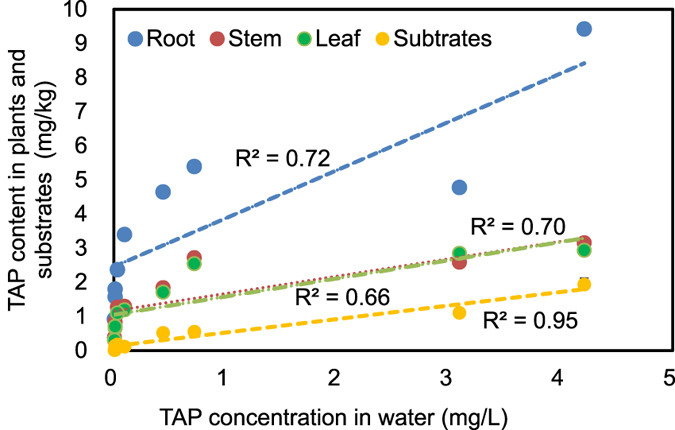
The correlation between TAP content in tissues of *Canna indica* and that in the substrates, as well as the TAP concentration in water from the sampling ports of the horizontal flow constructed wetlands (HSCWs) fed with mean influent TAP concentrations of 0.12 ± 0.04 mg L^−1^ (CW1), 0.79 ± 0.29 mg L^−1^ (CW2) and 3.96 ± 1.17 mg L^−1^ (CW3) at the hydraulic loading of 100 mm d^−1^.

The contents of TAP accumulated in ceramsites gathered from the front, middle and rear regions of the HSCWs are shown in Fig. 4 (n = 3). The TAP content in the substrates gradually declined along the flow direction, with the highest levels of 0.11 ± 0.04 mg kg^−1^ (CW1), 0.52 ± 0.02 mg kg^−1^ (CW2) and 1.94 ± 0.18 mg kg^−1^ (CW3) observed at the front regions. A two-way ANOVA reveals that both the influent TAP concentration as well as the distance along the flow direction had significant effects on the TAP content in substrates (P < 0.01). The TAP content in substrates increased with increasing influent TAP concentrations, with maximum values of 1.93 ± 0.18 mg kg^−1^ (front), 1.11 ± 0.08 mg kg^−1^ (middle) and 0.56 ± 0.06 mg kg^−1^ (rear) in CW3. Similar to plants, the TAP content in the substrates was linearly correlated with the TAP concentration in water along the flow path (Fig. 5, r^2^ = 0.95, P < 0.001).

The amount of TAP accumulated in plants and ceramsites increased with the influent TAP concentration; in addition, the TAP in ceramsites was correlated with the total of removed TAP quantity. The contributions of the TAP accumulation in plants and substrates to the total TAP removal were calculated based on the TAP mass balance (Table 1). The mean percentages of the accumulation in plants and substrates for the three CWs with respect to the total TAP removal were only 0.035 ± 0.034% and 4.33 ± 0.43%, indicating that over 95% of TAP removal from each of the systems was contributed through other mechanisms.

**Table 1:** The TAP accumulation in plants and substrates from the horizontal flow constructed wetlands (HSCWs) and their contributions to the total TAP removal.

	TAP amount removed (mg)			Contribution to total TAP removal (%)		
	Total amount removed	Accumulated in plants	Accumulated in ceramsites	Accumulated in plants	Accumulated in ceramsites	Other methods
CW1	447.17	0.32	17.19	0.07	3.84	96.09
CW2	2018.52	0.50	94.28	0.02	4.67	95.30
CW3	8890.56	0.66	397.65	0.01	4.47	95.52
Mean	3785.42	0.47	169.71	0.03	4.33	95.63

## Discussion

### Removal efficiency of TAP

The HSCWs were highly effective in the removal of TAP, the efficiencies of which remained stable when the influent concentration was below 1 mg L^−1^ (Fig. 1) in comparison with the removal efficiencies for pesticides reported in previous studies regarding CWs, which varied between 25% and 97%, and was >60% in the majority of the cases^9^. Considering that the influent pesticide concentrations in most of the applied studies were considerably lower than that in our study, e.g., the azinphos-methyl concentration from agricultural runoff was reported to range between 0.02 and 0.85 µg L^−1^ ^13^, it can be predicted that HSCWs have comparable or higher efficiencies with regard to the realistic treatment of TAP contaminated water. The removal efficiency is complex, and it is affected by many factors, including the hydraulic conditions of the wetlands^14^. Theoretically, with an increasing HLR, namely, with a reduction in the hydrological retention time (HRT), the removal efficiency declines^15, 16^. However, a further decrease of the removal efficiency was not evident at an HLR of 200 mm d^−1^ in our study when the influent TAP concentration was low (<1 mg L^−1^), which had been similarly reported for metolachlor by Stearman *et al*.^17^. Realistically, the TAP load, which can be obtained by multiplying the HLR and the influent TAP concentration, is likely a more proper parameter for discussion within CW studies.

Isopleth maps demonstrate that the major TAP removal zone was limited to the front half section of the CWs (Fig. 2), but extended to the middle and rear regions when the influent concentration increased, which could partially explain the high removal efficiency at low influent concentrations as well as the lower removal efficiency for CW3. Therefore, the length of the CWs and the influent TAP concentration should be considered during the design of CWs to properly treat TAP contaminated water. The longitudinal variation in pesticide concentrations along the flow in the CWs is also affected by hydraulic conditions^9, 18^. Increased shear velocities and turbulent mixing enhance the longitudinal dispersion of fluid momentum^19^. HLR was proven to influence the longitudinal distribution of TAP in our study, wherein the contour lines were ‘pushed’ ahead further at an HLR of 200 mm d^−1^ relative to that at100 mm d^−1^. Moreover, higher TAP concentrations than in the influent were observed between 20 cm and 40 cm from the distribution wall, indicating the probable presence of a mildly clogged flow in CW3 at 100 mm d^−1^ (Fig. 2). At the higher HLR (200 mm d^−1^), the hydraulic conductivity was improved, thereby providing a reasonable explanation of the TAP distribution.

### Accumulation in plants and substrates

The TAP concentration in the roots of *C. indica* was particularly higher than in other tissues (Fig. 3), which is consistent with the general pattern of accumulation of organic compounds in plants^20, 21^. The accumulation of TAP in plant tissues is correlated with the TAP content laterally along the direction of flow, descending from the front to the middle and rear section; similarly, previous research has shown the decline of pharmaceutical and personal care products in plant tissues from the inflow to the outflow of a CW^22, 23^.

The TAP accumulation in substrates was associated with the absorption and desorption kinetics of TAP that accompanies the first-order kinetics mode^24^ (Fig. 4). The close correlation between the TAP content in substrates and the TAP concentration in the water along the flow (R² = 0.95) partially suggests that the absorption and degradation capacity of ceramsites outperformed the influent TAP concentrations (Fig. 5).

### Removal pathways and their contributions

Generally, certain organic compounds, e.g., TAP, demonstrate a moderate range in their octanol-water partition coefficient (0.5 to 3.5), and can thus be easily absorbed and translocated into plants^1, 25^. However, due to some protection mechanisms meant to avoid excessive accumulation, e.g., phytodegradation and phytovolatilization^22^, *C. indica* did not accumulate TAP in proportion with the influent TAP concentration; meanwhile the contribution of substrates demonstrated a correlative rise with the influent TAP content. The accumulation in plants and substrates only contributed 3.91–4.69% of the removal of TAP (Table 1), indicating that the majority of the imported TAP was degraded through other mechanisms. The major abiotic degradation mechanisms include hydrolysis, photodegradation, and redox reactions^24^, but these processes only contribute a combined TAP removal of 9% within a hydroponic system containing *C. indica* ^10^. Therefore, the majority of TAP input is degraded within a CW system through biotic mechanisms.

Although TAP accumulation in plants accounts for negligible percentages of the total removal (<0.1%), plant tissues play important roles in the treatment of pesticides in CWs. Vegetated CW or hydroponic systems generally remove more pesticides, such as TAP, simazine and metolachlor, than non-vegetated systems^7, 26^. Moreover, the contribution of plants to the removal of TAP has been estimated at 74% in a hydroponic system through a comparison experiment^10^. Apart from biodegradation and phytovolatilization, plants also contribute to the removal of pesticides through rhizodegradation, wherein microbial activities are enhanced either in the rhizosphere and/or through the secretion of root exudates for the degradation of organic pollutants^11^. Toyama *et al*.^27^ reported that the interactions between Mycobacterium and root exudate could accelerate the biodegradation of pyrene and benzo [a] pyrene in the rhizosphere of *Phragmites australis*. Reports have also confirmed that the production and release of urease and phosphatase by *C. indica* was an important mechanism for TAP degradation^10, 28^.

The approaches through which substrates contribute to the purification of organic contaminants include direct adsorption and sedimentation, as well as indirect methods, such as enhancing the formation of biofilms by providing a favourable environment for microorganisms. The contribution of TAP accumulation in ceramsites was less than 5% (Table 1), suggesting that adsorption did not represent the major removal pathway in the substrates. Most of the removal of TAP was achieved by microbial degradation^3, 10, 29, 30^, and the substrates in CWs provide various and suitable circumstances for the development of microbial populations. Therefore, the roles of substrates, plants and microbes and their interactions should be considered with respect to the design and application of CWs for pesticide removal.

### Conclusions

This study has proven that HSCWs have high TAP removal efficiencies of over 90% when the influent TAP concentration is below 1 mg L^−1^. The majority of TAP removal occurs in the front half section of the HSCWs, while the primary purification area will move forward coincident with increasing influent TAP concentrations. TAP removal within the HSCWs was achieved by phytoaccumulation (0.03%), absorption through substrates (4.33%) and other mechanisms (95.63%). Most of the removed TAP by the HSCWs was likely eliminated by the joint contribution to degradation by plants and microbes. Therefore, the manner in which the interactions among plants, substrates and microbes is strengthened, as well as for the optimization of the longitudinal scale and HLR of the system, should be taken into account with respect to the design and application of CWs for the treatment of pesticide contaminated water.

## Materials and Methods

### Settings and operations of the constructed wetlands

**Figure 6:**
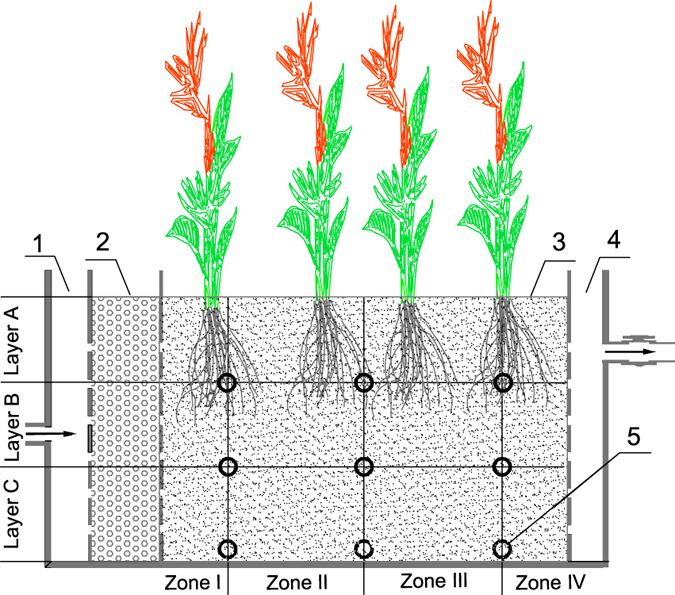
Longitudinal diagram of the horizontal subsurface flow constructed wetlands (HSCWs). (1) Inlet chamber; (2) distribution chamber; (3) growth chamber; (4) outlet chamber; (5) water sampling port. Arrows demonstrate the flow direction.

Three pilot-scale HSCW units were established in a greenhouse that was not equipped with air conditioning or heating at the Tongji University campus using polyvinyl chloride (PVC) containers with sizes of 120 × 40 × 70 cm (length × width × height). Each unit consisted of four chambers as shown in Fig. 6: an inlet chamber (8 × 40 × 70 cm), a distribution chamber (15 × 40 × 70 cm), a growth chamber (90 × 40 × 70 cm) and an outlet chamber (7 × 40 × 70 cm). The distribution chamber served as a distributor of inflow water from the inlet chamber and was filled with quartz gravel (with diameters of 3–8 mm). The growth chamber was the primary treatment section of the wetland; it was filled with ceramsite (with a diameter of 1–3 mm) and planted with *Canna indica* at a density of 20 plants m^−2^. Nine water sampling ports were installed in a grid-like fashion along the sidewall of the growth chamber with an interval of 30 cm wide and 20 cm high relative to one another. The substrate in the growth chamber was 50 cm thick and was horizontally divided by the three vertical columns of sampling ports into four zones as illustrated in Fig. 6 (Zones I, II, III and IV). The substrate was also vertically divided into three layers (A, B and C) from the top to the bottom.

The synthetic influent was prepared using tap water with 20 mg L^−1^ sucrose, 1.04 mg L^−1^ monopotassium phosphate and 2.48 mg L^−1^ urea, and it was stocked in three 0.5 m^3^ tanks. The average pH, dissolved oxygen (DO), chemical oxygen demand (COD) and total nitrogen (TN) in the tanks were 7.1 ± 0.3, 4.1 ± 1.5, 104.0 ± 16.5 and 3.6 ± 1.0 mg L^−1^, respectively. Prior to initiating the trial, the CWs were fed TAP-free influent for approximately three months to stabilize the systems. Afterwards, TAP diluted in methanol was added into the stock tanks to reach three different TAP concentrations (0.12 ± 0.04, 0.79 ± 0.29 and 3.96 ± 1.17 mg L^−1^). The influent with added TAP was pumped using peristaltic pumps into the three wetland units (CW1, CW2 and CW3, respectively), and was subsequently collected through standpipes installed in the outlet chambers.

The trials for removing the TAP were conducted at a hydraulic loading rate (HLR) of 100 mm d^−1^ from September 2012 to March 2013, and at 200 mm d^−1^ from April 2013 to August 2013. The average annual temperature during the experiment was 16.5 °C; the lowest average monthly temperature of 3.3 °C was observed in January and the highest average monthly temperature of 27.8 °C was observed in August.

### Analyses of TAP in water, substrates and plants

To test the TAP removal at different influent TAP concentrations for the two HLRs, inflow and outflow water samples were collected every month. In addition, to investigate the distribution of TAP in substrates to indicate the TAP removal tendency along the direction of flow, water samples from the nine ports (Fig. 6) were also obtained. Each sample was filtrated through a 0.45 μm filter membrane, and 200 mL of the filtrated sample was passed through a solid phase extraction (SPE) micro column at a rate of 3–5 mL min^−1^. The SPE micro column was flushed with 5 mL ultrapure water, and was subsequently suctioned with steady airflow for 10 min, and finally eluted with 5 mL acetonitrile. The eluant was collected and stored in a refrigerator at −20 °C for the TAP detection.

To evaluate the contributions of plants and substrates to the removal of TAP in the CWs, plant and substrate samples were collected from the front, middle and rear zones of the growth chamber (20, 60 and 85 cm from the inlet-side of the growth chamber, respectively) at an HLR of 100 mm d^−1^ in December of 2012. The plants were uprooted, cleaned with water and were subsequently separated into roots, stems and leaves and were then, the plants were freeze-dried, smashed into powder and sieved through a 200-mesh sieve for the TAP determination. A plant powder of 0.50 g was extracted using 50 mL dichloromethane using an application of ultrasonication for 20 min. After the solution was clarified, the supernatant was collected, condensed through rotary evaporation and dissolved with 5 mL methanol. The extract was filtrated through an SPE micro column and eluted by 5 mL acetonitrile. The eluent was collected and stored in a refrigerator at −20 °C for the TAP detection.

The ceramsite samples were collected from the plant root zone at the same locations as the plant samples. After the removal of impurities, such as plant roots, the ceramsites were air-dried, ground up, and then sieved through a 200-mesh sieve. A substrate sample of 5.0 g was extracted using 25 mL dichloromethane in a vibrator at 30 °C for 30 min. The following procedures were consistent with those used for the plant samples.

The TAP concentration of the extracts was measured by an HPLC (1200 serial, Agilent, Palo Alto, California, USA), which was furnished with a Diode Array Detector. A Water Symmetry C18 column (5 μm, 4.6 mm × 250 mm inner diameter, Waters, USA) was used for the separation. The analytes were eluted with a water-methanol (2:8, V/V) mixture at a flow rate of 1.00 mL/min. UV detection was performed at 246 nm for TAP, the retention time for which was 5.7 min with a column temperature of 30 °C.

A spike recovery study of TAP was conducted, and then the coefficients of recovery from the water sample, plant sample and substrate sample were 95.7 ± 4.7%, 88.8 ± 8.1% and 86.5 ± 7.2%, respectively, which correlate with the detection of TAP residue in the environment.

### Data analysis

The TAP mass balance in an HSCW was calculated using the following equations:

1 $${{\rm{R}}}_{{\rm{total}}}={{\rm{A}}}_{{\rm{p}}}+{{\rm{A}}}_{{\rm{s}}}+{{\rm{R}}}_{{\rm{other}}}$$

2 $${{\rm{R}}}_{{\rm{total}}}=({{\rm{C}}}_{{\rm{in}}}-{{\rm{C}}}_{{\rm{out}}})\times {{\rm{Q}}}_{{\rm{in}}}$$

3 $${{\rm{A}}}_{{\rm{p}}}={{\rm{C}}}_{{\rm{p}}}\times {{\rm{Q}}}_{{\rm{p}}}$$

4 $${{\rm{A}}}_{{\rm{s}}}={{\rm{C}}}_{{\rm{s}}}\times {{\rm{Q}}}_{{\rm{s}}}$$

where R_total_ denotes the total amount of TAP removed by the HSCW during the experimental period. A_p_ and A_s_ represent the TAP accumulated in the plants and substrates, respectively. R_other_ is the TAP quantity removed through other mechanisms. C_in_ and C_out_ represent the average TAP concentration (mg L^−1^) of the inflow and outflow. Q_in_ denotes the total inflow volume (L) during the experiment. Q_p_ and C_p_ are the plant biomass (g DW) and TAP concentration accumulated in plants (μg g^−1^ DW). Q_s_ and C_s_ are the substrate mass (g) and TAP concentration accumulated in substrates (mg kg^−1^), respectively.

To quantify the removal tendency of TAP in HSCWs, an isopleth map of TAP concentrations along the flow was generated utilizing gridding and contouring functions in Matlab 2016b (MathWorks, Natick, MA USA), using the TAP concentrations of the inflow and outflow, as well as of the water samples from the nine sampling ports.

A two-way ANOVA was performed using SPSS Statistics19.0 (SPSS Inc., Chicago, IL USA) to evaluate the effects of the influent TAP concentration and HLR on the TAP removal efficiency. A post hoc test was used to determine the pairwise differences between influent TAP levels. Furthermore, the correlation between the TAP contents in plant tissues and substrates and the TAP concentration at the locations where the plants and substrates were collected was subsequently tested. The normality of the data and homogeneity of variances was ensured before the ANOVA tests were performed. P values of less than 0.05 were considered to be statistically significant.

P-values of less than 0.05 were considered to be statistically significant.
